# Proposal to the Indian Psychiatric Society for adopting a specialty section on addiction medicine (alcohol and other substance abuse)

**DOI:** 10.4103/0019-5545.37669

**Published:** 2007

**Authors:** Vivek Benegal, Alok Bajpai, Debasish Basu, Neelam Bohra, Sudipto Chatterji, RB Galgali, DS Goel, Mohan K Isaac, Venugopal Jhanwar, Rajkumar Lenin, PM Madhavan, AK Mittal, E Mohandas, Thyloth Murali, Pratima Murthy, Rajesh Nagpal, S Nambi, C Ram Subramaniam, Shubhangi Parkar, Prasad Rao, MS Reddy, Alok Sarin, TP Sudhakar, BM Tripathi, Matthew Varghese

**Affiliations:** Members, Indian Psychiatric Society

The Indian Psychiatric Society, laudably, has a few special subsections catering to the advance and development of certain areas of mental-health care. These are devoted to areas or disciplines which are special to the health of the people of India but for historical reasons have not received the attention they deserve and therefore require affirmative action and special nurturing.

This document outlines the need to adopt a similar approach to persons with alcohol- and drug-related problems in India and to create a specialty section on Addiction Medicine, under the purview of the Indian Psychiatric Society.

## BACKGROUND

### Alcohol, tobacco and other substance abuse affects a disproportionately large section of people in India

The current prevalence rates among male adults (i.e., subjects who had used within the last 1 month) according to the NHS are as follows: alcohol 21.4% [62.5 million]; cannabis 3.0% [8.7 million]; opiates 0.7% [2 million]; any illicit drug 3.6% [10.5 million]. These numbers, when applied to the total Indian population of 102.7 crores in 2001, provide prevalence rates of 60/1000, 8/1000 and 2/1000 population respectively.[1]

Around 25% of current users were ‘dependent’ users. Dependent users as a proportion of current users were 17% for alcohol, 26% for cannabis and 22% for opiates. A meta-analysis by Reddy and Chandrashekar[2] revealed an overall prevalence of alcohol dependence to be 6.9/1000 for India, with urban and rural rates of 5.8/1000 and 7.3/1000 population. The rates among men and women were 11.9 and 1.7 respectively. It is also well recognized that the prevalence rates are not uniform and vary widely within the country. The prevalence rates for alcohol- and other substance-use disorders are much higher – for example, in some of the northeastern states (prevalence of alcohol use was above 65% among men and above 50% in Arunachal Pradesh).[1]

The figures for prevalence of substance use may at first appear deceptively small, especially the figures for alcohol use, when compared to global estimates; but the absolute numbers are huge. Also, it is increasingly apparent that the health burden, as well as the social cost attributable to alcohol misuse, is due in greater measure to persons with hazardous use than to persons with dependent use. Hazardous use has been estimated at over 80 and 55% of all male and female users respectively.[3]

There is no such comfort of ‘low prevalence’ as far as tobacco use is concerned. There are currently about 240 million tobacco users aged 15 years and above (195 million male users and 45 million female users) in India.[4]

Prescription drug abuse, especially of sedatives/ hypnotics [0.3 million as per the NHSDAA, 2003], is a growing problem, which has gone largely unrecognized in India. Media reports suggest rising abuse of stimulants by students as awakening pills (for exams, etc.) or as recreational agents in ‘rave’ parties. Then there is the largely unstudied phenomenon of inhalant abuse, which is widely prevalent among ‘hidden populations’ such as street children.

### The prevalence is more than that of all severe mental disorders combined

The gravity of the problem of substance-use disorders can be highlighted by comparing their prevalence with that of other psychiatric disorders. Recent studies have generated all-India prevalence rates for all severe mental disorders of 58/1000[2] to 73/1000.[5]

### The health burden attributable to substance misuse is inordinately high

The impact of a health problem should not be gauged merely by its prevalence but by the health burden and social cost that it wreaks on society. Unfortunately, the focus of the professional community, as well as public scrutiny, has been primarily on the phenomenon of dependence or ‘addiction’ in the individual client. In this context, the excessive and problematic use of a drug that is hard for the individual to control becomes the target. The problems caused by, and associated with, substance use are far broader. They include problems partly or wholly caused by intoxication, such as injuries and some acute illnesses; long-term effects on health of a pattern of substance use that does not otherwise disrupt social functioning; acute and chronic health problems caused by unsafe ways of using drugs otherwise unrelated to pharmacological effects (e.g., using dirty needles); impacts on other people (e.g., secondhand smoke); and impacts of the criminal justice system on people engaging in illegal behavior.

### Alcohol- and tobacco-related problems in India

Hospital admission rates due to the adverse effects of alcohol consumption are disproportionately high. Several studies indicate that nearly 20–30% of admissions/ consultations are due to alcohol-related problems (direct or indirect) in different health-care settings but are under-recognized by primary-care physicians.[6,7] Alcohol misuse has been implicated in over 20% of traumatic brain injuries[8] and 60% of all injuries reporting to emergency rooms.[9] It has a disproportionately high association with deliberate self-harm,[10] high-risk sexual behavior,[11] HIV infection, tuberculosis,[12] esophageal cancer,[13] liver disease[14] and duodenal ulcer.[15] A recent estimate from surveillance of major noncommunicable diseases in India placed the burden due to alcohol as the *numero uno* among all noncommunicable disorders.[16]

There are 700,000 deaths per year due to smoking and 800,000 to 900,000 per year due to all forms of tobacco use/ exposure in India. For the next 20 years, there will be a faster trajectory of rise in tobacco-related deaths. Many of the deaths (>50%) will occur below 70 years of age.[17]

The social cost due to alcohol- and tobacco-related problems is prohibitively high.

Alcohol misuse wreaks a high social cost. In addition to the health costs, there are indirect costs linked to a wide variety of social implications (family disruption, marital disharmony, impact on development of children, deprivation of the family, absenteeism and industrial loss, crime and violence, etc.).[18–20] The social cost attributable to alcohol use, extrapolated to the entire country, was estimated at Rs. 244 billion for the year 2003–04; whereas the total excise revenues generated from alcohol manufacture and sales was Rs. 216 billion.[20] An earlier study from Karnataka estimated that monetizable direct and indirect costs attributable to people with alcohol dependence alone were more than three times the profits from alcohol taxation and several times more than the annual health budget of that state.[21]

The cost of tobacco-attributable burden of just three groups of diseases – cancer, heart disease and lung disease – was estimated at Rs. 308.33 billion in 2002-2003.[17]

### Alcohol and substance dependence/ abuse is very often comorbid with other communicable and noncommunicable disorders

Axis 1 psychiatric disorders (psychosis, mood disorders, anxiety disorders) and substance abuse co-occur more frequently than can be explained by chance alone. Patients with schizophrenia are 4.6 times more likely to have substance-use disorders than persons without mental illness (3 times higher for alcohol, 6 times higher for other illicit drugs).[22] While it has been previously commonly held that substance-use comorbidity in schizophrenia represents self-medication, an attempt by patients to alleviate adverse positive and negative symptoms, cognitive impairment or medication side effects; recent advances suggest that increased vulnerability to addictive behavior may reflect the impact of the neuropathology of schizophrenia on the neural circuitry mediating drug reward and reinforcement. Thus, schizophrenic patients may have a predilection for addictive behavior as a primary disease symptom in parallel to, and in many cases independent from, their other symptoms. There is also early evidence that the pathology in the neurobiological substrates that underlie mood and anxiety disorders also subserves the predisposition to substance-use problems.[23]

Substance abuse, especially of alcohol and tobacco, and a spectrum of high-risk behaviors, which often go together, are common risk factors for most of the noncommunicable disorders (like hypertension, diabetes, heart disease, cancer), which are assuming increasingly larger proportions of the health burden on the Indian society.[3]

Drug abuse and addiction also have tremendous implications for the health of the public, since drug use, directly or indirectly, is now seen as a major vector for the transmission of many serious infectious diseases, particularly HIV/ AIDS, hepatitis and tuberculosis; and is instrumental in one of the largest sources of mortality and morbidity in India, namely, road traffic accidents and violence.[20]

### Substance use disorders, especially involving alcohol and tobacco, are grave public health problems

It obviously follows from the above statements that substance-use disorders need to be recognized as public health problems and that there is urgency for psychiatrists to liaise with other medical specialties, the public health delivery system, as well as organizations working in the area of development. As specialists in bio-behavioral disorders, psychiatrists are in an ideal position to do so, and the problem requires the involvement of diverse medical professionals.

Here, one needs to exercise caution and steer clear of the ‘me-too’ syndrome, wherein one creates a specialized discipline which clamors for an independent share of limited health-care resources. It would be prudent for the psychiatrist community to work in tandem with, and to juxtapose its strength and skill-sets with, the existing networks and delivery systems for communicable and noncommunicable disorders.

Addiction medication is an area where the psychiatrist can fruitfully work in tandem with other medical specialists, and in some ways this will be a good medium to get psychiatry into the mainstream. So the advantage can be mutual.

Alcohol and tobacco use are also strongly affected by the economic and political compulsions of the day. One needs to only look at the recent imbroglio over the issue of scary pictures of consequences of tobacco use on cigarette packs and the subsequent backtracking by the Ministry of Health in the face of pressure from the tobacco lobby.[24] Similarly, the pursuit of a rational alcohol policy has been vitiated by economic compulsions of the states and the misdirected coercion of market economics. Thus, there is very little debate on the recent active moves of the grape-growers' lobby to have the Finance Ministry re-designate wine as an agricultural product.

Macro-level interventions in these domains are likely to be more effective and cost-effective. Intensive advocacy at several levels should therefore be a part of any endeavor in this area. The Framework Convention on Tobacco Control (FCTC) is a classic example and could provide the template for such initiatives at the national/ international level. The negative economics of addictions (e.g., the social costs of alcohol and tobacco overshadowing tax revenues from these commodities) must be carefully articulated as these are potent advocacy tools. It is also necessary to examine and publicize the cost-effectiveness of interventions to control substance abuse. The role of the industry and its vested interests (e.g., the powerful liquor and tobacco lobbies), as well as the complicity of medical professionals and pharmacists (as in benzodiazepine and other over-the-counter–medication misuse), should also be a focus of any remedial strategies.

The psychiatric community is specially equipped to provide leadership to such activities and bring to bear the valuable experiences gained from the successes and mistakes of the community psychiatry movement in India.

### Substance-use disorders constitute a large proportion of the clinical load in both private and public-health psychiatric practice

Substance-use disorders already form more than 20% of the case load of psychiatric practitioners, often, as noted above, inextricably associated with mood, anxiety and psychotic disorders. However, as a recent piquant exchange on a popular Indian psychiatrists' bulletin board revealed, many psychiatrists are loath to take on clients with substance-use disorders, preferring to refer them on to a very short list of colleagues who will see them. This is often related to a feeling of helplessness and frustration faced with the usual picture of recidivism that typifies the natural history of persons with alcohol and drug disorders.

### Practitioners have an attitude of therapeutic nihilism towards addiction medicine and the addicted patient

These experiences translate into an attitude of therapeutic nihilism, which unfortunately is widely prevalent across the medical community. There are several factors which appear to contribute to this way of thinking:

#### Lack of education about alcohol and substance abuse:

Only a very small portion of the undergraduate medical syllabus, and even the postgraduate psychiatry syllabus, is devoted to this area, and it is limited only to the medical complications of alcoholism.

#### Negative attitudes about alcoholism:

Physicians and psychiatrists in training often see late-stage alcoholics, who often evoke feelings of aversion, hostility and helplessness. Some physicians tend to see alcoholics and drug addicts as bad or morally weak.

#### Discomfort with related social issues:

Since alcoholism involves not just medical issues but significant psycho-social issues also, many physicians are uncomfortable dealing with it.

#### Pessimism about treatment:

There are many physicians who feel that addiction is not treatable. Part of the helplessness and pessimism occurs because of the experience with late-stage problem drinkers, where the treatment is not always successful. The physician must remember that early detection of, and intervention to resolve, alcohol-, tobacco- and other substance-related problems offers the best results.

It is not surprising, then, that the field of addiction psychiatry currently suffers the same pariah status that psychiatry was once ‘favored’ with among other medical professionals. The drug rehabilitation centers of the early 21^st^ century often serve the same function that the leprosaria and asylums of the early 20^th^ century did: seclusion and restraint!

### Leadership in the field of advocacy and policy making or legislation has been ceded to nonmedical, nonpsychiatric bodies

To a large extent, this pessimism also arises from the fact that till lately, there have not been too many effective treatments. Here one must invoke the late Abraham Maslow, who famously said: “If your only tool is a hammer, all your problems look like nails!” For far too long, treatment strategies have been limited by a *single-hammer* model dominated by an abstinence paradigm and equipped with limited behavioral-change strategies.

Unconscionably, physicians in frustration have attempted to curb the uncontrollable behaviors of persons with drug and alcohol problems with antipsychotics and electroconvulsive therapies. This has only served to alienate them from this client group. One has only to attend a survivors' group meeting to realize the hostility that psychiatrists in general are viewed with.

Not surprisingly, the onus of treating these clients has shifted either to survivor groups like the Alcoholics Anonymous and Narcotics Anonymous; or to nonmedical, nonpsychiatric rehabilitation agencies. Unsurprisingly, the leadership in the field of advocacy and policy making or legislation in the area has also been ceded to nonmedical, nonpsychiatric bodies.

One needs to mention here that the standards of treatment of clients with addictive disorders are variable, at best. The various agencies involved differ widely in the methods they utilize and in a mostly unregulated field; there are numerous instances of clients being subjected to practices which are questionable, at best, and often highly unethical and dangerous.

It is incumbent upon the members of the society to use their collective influence to bring some regulation and standardization of procedures into the field, being guided by evidence. A good point to start from is the documentation on treatment protocols that the society has previously generated, as well as the guidelines generated by the ministries of Health and Social Justice and Welfare.

### Addictive disorders are bio-behavioral disorders which are strongly impacted by economic and political influences

However, one needs to assert here that psychiatrists, because of their training as bio-behavioral specialists, are best positioned to coordinate the management of this array of problems. Increasingly, these recurrent and relapsing illnesses are being recognized to be a resultant of the unfortunate pairing of substance use with a neurobiological diathesis modulated by environmental influences. Recent studies have demonstrated that susceptible individuals have smaller volumes in brain areas critical for attention, learning, judgment, reward and motivation (possibly due to a maturational lag affecting myelination). This may result in a trait of central nervous system hyperexcitability, usually manifest as a spectrum of externalizing behaviors marked by compromised impulse control, high novelty-seeking or sensation-seeking behavior, increased response to reward, a tendency to pay more attention to immediate rewards and neglect long-term outcomes when making decisions, reduced response to punishment and a disregard for social norms. This is associated with heightened reinforcement from alcohol and drugs coupled with impaired early warning of intoxication. A significant portion of this research has come out of India! In effect then, drug dependence, in large part, may be the epiphenomenon of, or a variable expression of, a generalized disinhibitory complex, which has been hypothesized to predispose to mood disorder, anxiety disorder and perhaps schizophrenia! Susceptible individuals may be self-medicating themselves and initially even successfully reversing, or compensating for, their low motivational responses to normal rewards.[25]

This is important to assert; because, for far too long, despite the lip service paid to the medical and bio-psycho-social model of alcohol and other substance dependence, practitioners have rarely been able to distance themselves from the prevalent social discourse which regards drug users through the lenses of the moral model as ‘losers – who are too weak to stop and who deserve what they get!’

Also, the growing literature on the long-lasting neuro-adaptations consequent to prolonged exposure to substances of abuse is beginning to delineate the neuropathology that gives rise to the characteristic behavioral changes which account for the recurring and relapsing nature of the illness. Slowly, these advances from the laboratory benches are being translated into changes in behavioral and pharmacological strategies for more efficacious treatment. There are recent reports on the efficacy of newer pharmacological agents and audits of individual and population-level interventions which are more efficacious than earlier remedies.[26–28] A special and concerted effort is required to test and document these interventions and make them broad based, while at the same time bringing them into public notice.

And the community of psychiatrists is well positioned to spearhead these efforts at advancing the study of the bio-behavioral determinants of this array of disorders and incorporating the opportunities derived from research into day-to-day practice.

### Addiction medicine is a prominent and active subsection in most international psychiatric bodies

It is instructive to look at the status of addiction medicine, within the structure of other international psychiatric bodies. In the USA, for example, doctors focusing on addiction medicine are medical specialists who focus on addictive disease and have had special study and training focusing on the prevention and treatment of such diseases. There are two routes to specialization in the addiction field: one via a psychiatric pathway and the other outside of psychiatry. The American Society of Addiction Medicine notes that approximately 40% of its members are psychiatrists, while the remainder has received medical training in other fields.

The facts stated above underline the urgent need for the Indian Psychiatric Society to constitute a specialty section (with appropriate regional representation) devoted to Addiction Medicine, within its ambit.

## AIMS AND OBJECTIVES

### The specialty section on Addiction Medicine will be required to:

- Promote and advance the bio-psycho-social contributions to the science and practice of Addiction Medicine in all its different manifestations;
- Promote a] the improvement of the health of persons directly or indirectly impacted by alcohol- and other substance-related problems and b] prevention, control, treatment and relief of all alcohol- and other substance-related problems, as well as conditions which constitute high risk for potential drug- and alcohol-related problems. This involves encouraging and enabling members of the society to explore, extend and pursue ‘evidence-based’ treatment strategies and prevention methods. This includes liaison with other stakeholders, including, but not limited to, a] governmental agencies overseeing health, social justice and welfare, finance and human development; b] nongovernmental agencies involved in direct care of persons with substance abuse, as well as agencies whose developmental programs are impacted unfavorably by substance-abuse problems directly or indirectly, affecting their beneficiaries; and c] other medical specialties and primary health-care providers. This also requires sharing the expertise of members of the society and other professionals involved in diverse fields such as school mental health, community-based rehabilitation, epidemiology, drug trials, maintaining disease registries, to name just a few;
- Actively engage in advocacy in order to influence policy making and legislation, keeping in mind that substance-use disorders and their consequences constitute a grave public health disorder which imposes an unacceptably severe burden on the health and well-being of the people of India. This, above all, requires consistent accumulation and dissemination of data on the economic, social and health costs of substance abuse to the Indian society; and on the evidence of cost-efficacy of appropriate intervention. There is emerging conviction that the risk factors and consequences of substance-use disorders are often common to, and frequently complicate, the development and outcome of a whole host of medical disorders and social ills. Synergizing with other stakeholders mentioned above, in sharing of methodologies, will strengthen each other's effort to mutually benefit both in terms of intervention impact as well as the collective bargaining power to advocate change. Such co-action will also reduce the competing and conflicting 'me-too' demands stemming from parallel agencies working towards similar ends, which overwhelm limited resources of finance and manpower and weaken the effectiveness of the discourse for change;
- Formulate and recommend standards of treatment and rehabilitation of persons or populations affected directly or indirectly by substance-use disorders. In addition, it will be necessary to obtain recommendations for syllabi and commission resources for the education and training of medical and nonmedical personnel involved in the field;
- Safeguard the interest of psychiatrists and fellow professionals engaged in the practice of Addiction Medicine in India and promote ethical standards in the practice of Addiction Medicine in India; and
- Promote research in the field of addiction science with a focus on translating the benefits of research to the bedside and propagate the use of evidence-based interventions in the daily delivery of mental-health care.

## SUGGESTED ACTIVITIES

1. A web portal, linked to the web page of the Indian Psychiatric Society, which will initially host information of relevance to the practice of Addiction Medicine in India, with direct and indirect links to published or unpublished scientific papers, databases, treatment protocols, available treatment agencies, online resources, etc.; and later, e-learning sites for online education and training.
2. Coordinating Continuing Medical Education (CME) pertaining to the aspect of the science and practice of Addiction Medicine, with special emphasis on underserved areas like the northeastern states, Andaman and Nicobar Islands, etc.
3. Enabling and commissioning position papers on the impact of substance abuse in India, with a view to influence public policy.
4. Engaging with relevant organizations to promote demand-reduction strategies, such as the police to prevent drinking and driving, primary health providers and emergency rooms for early detection and brief intervention.
5. Pursuing the aim of working to safeguard vulnerable populations, e.g., inoculating children and adolescents through skills training; sensitizing physicians and pharmacists to prevent over-the-counter drug abuse, especially for the elderly.
6. Examining the possibility of creating a national registry, using standardized data-entry protocols.

The proposal to constitute a specialty section on Addiction Medicine was first mooted by Dr. Varghese Punoose at the IPS South Zone CME program at Kodaikanal in 2007. It was seconded by Dr. M. S. Reddy and received enthusiastically by the IPS President, Dr. I. R. S. Reddy. This document is in continuation of that process; and we urge the Society to place the document before the appropriate body, to initiate the necessary processes so that the Society may further debate and hopefully set in motion the steps to give substance to this proposal.
